# Advantages of the multiple case series approach to the study of cognitive deficits in autism spectrum disorder

**DOI:** 10.1016/j.neuropsychologia.2009.06.028

**Published:** 2009-11

**Authors:** Karren J. Towgood, Julia D.I. Meuwese, Sam J. Gilbert, Martha S. Turner, Paul W. Burgess

**Affiliations:** Institute of Cognitive Neuroscience, University College London, United Kingdom

**Keywords:** Neuropsychology, Variability, Asperger, Functional re-organisation

## Abstract

In the neuropsychological case series approach, tasks are administered that tap different cognitive domains, and differences within rather than across individuals are the basis for theorising; each individual is effectively their own control. This approach is a mainstay of cognitive neuropsychology, and is particularly suited to the study of populations with heterogeneous deficits. However it has very rarely been applied to the study of cognitive differences in autism spectrum disorder (ASD). Here, we investigate whether this approach can yield information beyond that given by the typical group study method, when applied to an ASD population. Twenty-one high-functioning adult ASD participants and 22 IQ, age, and gender-matched control participants were administered a large battery of neuropsychological tests that would represent a typical neuropsychological assessment for neurological patients in the United Kingdom. The data were analysed using both group and single-case study methods. The group analysis revealed a limited number of deficits, principally on tests with a large executive function component, with no impairment in more routine abilities such as basic attending, language and perception. Single-case study analysis proved more fruitful revealing evidence of considerable variation in abilities both between and within ASD participants. Both sub-normal and supra-normal performance were observed, with the most defining feature of the ASD group being this variability. We conclude that the use of group-level analysis alone in the study of cognitive deficits in ASD risks missing cognitive characteristics that may be vitally important both theoretically and clinically, and even may be misleading because of averaging artifact.

## 1 Introduction

The acquired cognitive deficits experienced by neurological patients are heterogeneous, since no two brains are exactly alike, and the damage to them will differ from individual to individual, and individuals will differ greatly in their pre-morbid characteristics. This fact led to the adoption, principally through the 1980s, of the neuropsychological single-case design, and became a mainstay of the field of cognitive neuropsychology. The principal aim of this field was to build models of how the normal cognitive system is organised (as opposed to linking particular domains of cognition or their disorders to brain structures). This approach has now been used to extend our understanding of brain–behaviour relationships in virtually all domains of cognition, including perception, memory, language, reading and writing, numeracy, and executive function, and there are key papers using the single-case study methodology in each of these areas that have revolutionized our understanding of how the supporting brain systems are organised (see e.g. Shallice & Evans, 1978 for review). The neuropsychological single-case method should not be confused with the principally descriptive case studies that have traditionally appeared in, for instance, neurology and psychiatry (although these have their own value). Neuropsychological case study is an empirical procedure whose starting points are particular deficits or symptoms displayed, and a model of how the relevant cognitive systems are organised. The investigation then proceeds through a series of stages that seek to isolate the precise processing locus underpinning the impairment, excluding as many other possibilities and potential artifacts as possible. The conclusion of the investigation might be, in theoretically oriented work, a challenge to the underlying cognitive model, or in clinically oriented work, a better understanding of the causes of the symptom. There are many expositions on, and justifications of, this now widely accepted method (e.g. Caramazza, 1986; Caramazza & McCloskey, 1988; Coltheart, 1984; Shallice and Evans, 1978), and the approach has also been applied to the understanding of cognitive deficits in populations other than neurological ones (e.g. schizophrenia; Shallice et al., 1991).

Another condition where applying a single-case study approach may prove particularly enlightening is autism spectrum disorder (ASD). ASD is a neurodevelopmental disorder affecting approximately 1.0% of the population (Baird et al., 2006) and which presents with problems in social interaction, verbal and non-verbal communication difficulties and repetitive and stereotyped disturbances of behaviour.

Behavioural and genetic studies find that ASD is unlikely to involve just a single primary processing deficit and is more likely to be associated with a complex pattern of deficits across and within domains (Happe, Ronald, & Plomin, 2006; Minshew, Goldstein, & Siegel, 1997). In addition, Newschaffer, Fallin, and Lee (2002) report that the large variety of neuropathological changes and variability observed across patients suggest that ASD is etiologically heterogeneous. Consistent with this suggestion, recent neuroimaging studies indicate an abnormal pattern of functional specialization in the brains of participants with ASD (Gilbert, Meuwese, Towgood, Frith, & Burgess, 2009; Pierce, Muller, Ambrose, Allen, & Courchesne, 2001). If the processes by which distinct brain regions become specialized for specific functions are disrupted in ASD, this may lead to complex and idiosyncratic patterns of strengths and weaknesses in particular individuals (Gilbert et al., 2009). In this case, neuropsychological approaches are required that capture the variability in cognitive performance that is likely to accompany such atypical brain organisation and etiological heterogeneity.

Neuropsychological studies investigating ASD have typically used group study designs to investigate cognitive impairments. These studies have revealed that individuals present with an uneven pattern of cognitive strengths and weaknesses (Frith, 2003b). The cognitive deficits may include, but are not limited to, poor language comprehension, problems with working memory, failure to use context to support memory, poor memory for complex visual information, difficulties with planning and sequencing, difficulties with set shifting and a range of difficulties with complex attention. Strengths have also often been noted on tests of spatial reasoning and abstract problem-solving, on tests of rote memory and on tests of focused attention (Frith, 2003b, 2004).

However, there is considerable disagreement between neuropsychological studies. For example, Hill (2004) reviews the evidence on executive function in ASD and finds mixed results. Whilst findings regarding some domains of executive function, such as inhibition of a pre-potent response, have been found to be fairly consistent, findings within other components, such as planning and mental flexibility, have been much more inconsistent (e.g. Mari, Castiello, Marks, Marraffa, & Prior, 2003; Minshew, Goldstein, Muenz, & Payton, 1992; Ozonoff & Jensen, 1999). Similarly, support for the weak central coherence theory of autism is also equivocal (Happé & Frith, 2006). From their review of over 50 studies Happé and Frith concluded that whilst there have been some consistent findings, particularly with tasks such as the block design and the embedded figures tasks, other tasks, such as the Navon figures (Navon, 1977) and various visual illusion tasks have produced inconsistent and sometimes negative findings (e.g. Happe, 1996; Mottron, Burack, Stauder, & Robaey, 1999; Plaisted, Swettenham, & Rees, 1999; Ropar & Mitchell, 2001). Finally, a recent review by Willcutt, Sonuga-Barke, Nigg, and Sergeant (2008) also concluded that the neuropsychological etiologies of ASD are complex and multifactorial, with no single deficit sufficient to explain all cases.

Three main explanations can be offered for this observed disparity. Firstly, perhaps the basis for these inconsistent results is that the ASD diagnosis incorporates individuals who are highly variable in terms of their abilities and disabilities, or is composed of multiple distinct subgroups. This could in turn lead to significant population sampling differences across studies. Moreover there is the strong danger of an “averaging artifact” (Shallice and Evans, 1978), where the pattern detected at a group level does not describe well any single member of the group. A second possible explanation is that the ASD diagnosis does define a group of individuals with a distinct pattern of strengths and deficits, but the deficit is such that it can cause different results according to prima facie relatively minor changes in administration or task formats. An explanation of this kind might for example be that the ASD participants do not use social cues in the testing session as well as controls, or perhaps that they have attentional perturbations that mean slight task administration differences have major effects on performance. This detection problem would likely be exacerbated if no one study administered a full range of tests that might help characterise the nature of this problem. A third possible explanation is that ASD causes impairments in sensory and other processes (e.g. language comprehension, perception, short-term attention). These in turn may then cause unpredictable impairments in tests aimed at measuring higher level tasks (e.g. problem-solving), depending on the low-level features of specific tasks (e.g. stimulus materials). Since tests of these more lower level processes have typically not been administered in ASD studies, these deficits are potentially not detected.

The current study therefore applied both a group and multiple single-case series methodology using a comprehensive battery of neuropsychological tests to interpret the patterns of cognitive strengths and weaknesses in ASD. We tested the hypotheses that (i) observed deficits in higher order cognitive tasks in the ASD group are independent of deficits on lower level processes such as language comprehension, visual perception and short-term attention; (ii) patterns of deficit will vary from individual to individual within the ASD group; (iii) that this varying pattern of deficits across individuals demonstrated by the single-case approach is not revealed using traditional group study-type analysis; and (iv) the single-case study analysis will reveal strengths not detected in the traditional group study analysis. In order to be conservative in our hypothesis testing, we restricted our study to those ASD individuals performing in the high-functioning range on standardized intelligence tests; individual differences are likely to be smaller in a group of this type compared with a more heterogeneous sample.

## 2 Methods

### 2.1 Participant population

Baseline characteristics for the ASD and control participants are presented in Table 1. Participants were 21 adults (17 males, 4 females) with ASD, all of whom had a clinical diagnosis of Asperger's syndrome. Participants were selected if both their performance and verbal IQ quotients exceeded 85. IQ was measured with the full 11 subtest battery of the Wechsler Adult Intelligence Scale-Third Edition (WAIS-III; Wechsler, 1997).

ASD participants had all previously received a clinical diagnosis of ASD. All ASD participants were also administered the Autism Diagnostic Observation Scale (ADOS; Lord et al., 2000). Participants were included in the study regardless of whether or not they received an Autism/Autism Spectrum diagnosis on the ADOS. In addition, ASD symptom presentation was measured using the Autism Spectrum Quotient (AQ; Baron-Cohen, Wheelwright, Skinner, Martin, & Clubley, 2001). Results for the ADOS and AQ are presented in Table 1.

Twenty-two VIQ, PIQ, FSIQ, age and gender-matched controls were also included in the study. Independent *t*-tests (two-tailed) indicated no significant difference for age, gender, VIQ, PIQ and FSIQ. Moreover, *p* values for each of these variables were above the 0.50 mark recommended by Mervis and Klein-Tasman (2004) to show strong overlap between the distributions in each group.

### 2.2 Measures

A range of valid, reliable neuropsychological tests which are routinely used in standard United Kingdom clinical neuropsychological practice were selected to tap a broad range of general cognitive abilities as discussed in the following section. All tests were administered according to the procedures outlined in the relevant testing manuals and published papers and a fixed order of testing was used for all participants.

#### 2.2.1 Language

The ability to comprehend language and understand instructions was assessed using the de Renzi Token Test Shortened Version which employs tokens of three different colours and three different shapes, presented in a random array. Participants were administered 15 complex but abstract commands involving these tokens (Coughlan & Warrington, 1978). The McKenna Graded Naming Test (McKenna & Warrington, 1980), a 30 item test which assesses the ability to name line drawings of objects of graded difficulty, was also administered.

#### 2.2.2 Perception

Object and space perception abilities were assessed with the “Shape Detection”, “Incomplete Letters”, “Object Decision” and “Dot Counting” subtests from the Visual Object Space Perception Battery (VOSP; Warrington & James, 1991). The “Shape Detection” subtest simply measures participants’ ability to detect an “X” on a card with an all over speckled pattern, with half of the cards containing an embedded and degraded “X” and half not containing an “X”. On the “Incomplete Letters” subtest participants attempt to name a degraded letter of the alphabet. On the “Object Decision” subtest the participant is presented with 20 cards, each printed with four black shapes one of which is a silhouette of a real object and three of which are silhouettes of nonsense objects, and asked to name the real object. Finally on the “Dot Counting” subtest participants simply count the number of dots arranged on separate cards. Participants also completed the “Minimal Features” and “Overlapping Figures” subtests from the Birmingham Object Recognition Battery (BORB; Humphreys & Riddoch, 1993). In the “Minimal Features” subtest participants were presented with three different pictures on each of 25 trials. One was a picture of the target object taken from the standard viewpoint, one was a picture of the target object taken from an unusual viewpoint and the third was an object visually similar to the target object. The task of the participant was to select the two matching objects. In the “Overlapping Figures” subtest speeded identification of non-overlapping letters, geometric shapes and objects were compared to speeded identification of overlapping letters, geometric shapes and objects.

#### 2.2.3 Memory

Verbal and visual memory was assessed with the “Story Recall” and “Figure Copy” subtests from the Adult Memory and Information Processing Battery (AMIPB; Coughlan & Hollows, 1985). On the “Story Recall” subtest participants are first read a short story of the type you may hear on the television/radio or read in a newspaper and then immediately, and after a delay of approximately 30 min, asked to freely recall the story. On the “Figure Copy” subtest participants are presented with a complex geometrical figure to copy and after completing this copy immediately, and after a delay of approximately 30 min, asked to freely recall the design. Participants also completed the full Doors and People battery (Baddeley, Emslie, & Nimmo-Smith, 1994). The Doors and People battery consists of four subtests and is designed to assess visual and verbal learning, free recall and recognition. On the “People Test” participants are first required to learn four forename/surname pairs with the assistance of a coloured photograph and after a short delay are again asked to recall these names. In the “Doors Test” participants are presented with a set of 12 coloured photographs of doors to learn and then later presented with a forced choice recognition task. On the “Shapes Test” participants learn four geometrical designs by first copying them, and then are asked to recall these shapes immediately, and again after a short delay. Finally, on the “Names Test” participants are presented with 12 forename/surname pairs to learn and then later presented with a forced choice recognition task.

#### 2.2.4 Executive function

Executive function was assessed with the “Zoo Map” and “Six Elements” subtests from the Behavioural Assessment of Dysexecutive Syndrome (BADS; Wilson, Alderman, Burgess, Emslie, & Evans, 1996), the Hayling and Brixton (Burgess & Shallice, 1997), the “Proverbs” subtest from the Delis Kaplan Executive Function Scale (DKEFS; Delis, Kaplan, & Kramer, 2001), the Cognitive Estimation Test (CET; Shallice & Evans, 1978), the Modified Card Sorting Test (MCST; Nelson, 1976), Controlled Oral Word Fluency (COWA; Benton, 1968), Trail Making Test (Reitan, 1958), and the “Information Processing Speed Parts A and B” subtests from the AMIPB (Coughlan and Hollows, 1985).

From the BADS, the “Zoo Map” subtest assesses planning by asking participants to show how they would visit a series of designated locations on a copy of a map, whilst also following a number of specified rules. On the “Six Elements” subtest participants are required to plan and organise their time to complete at least some of each of six separate sub-tasks, whilst following predefined rules. The “Proverbs” subtest from the D-KEFS measures mental flexibility and the ability to think abstractly and consists of eight sayings each of which the participant must give a meaning for, first in a free inquiry format and second by selecting from four multiple choice options. The CET measures the ability to generate effective problem-solving strategies and participants are asked to estimate answers to 10 questions such as “what is the length of the average man's spine” and “how tall is the average English woman”. The MCST measures set shifting and response inhibition and requires participants to sort cards on one of three possible dimensions (colour, number, shape) according to an unspoken rule. After correctly sorting six cards, the participant must shift to sort the cards along another dimension and so forth until the completion of the test. The COWA measures verbal fluency and requires participants to verbally generate as many words as possible beginning with three letters of the alphabet, being in the case of the current study F, A and S. Participants are given a 60 s time limit for each letter and must obey certain rules. The “Trail Making Test” is a test of processing speed and set shifting and requires participants to draw lines connecting a series of 25 circles. In Part A participants must connect the numbers 1–25 placed randomly over an A4 page and in Part B participants connect the letters A-L and the numbers 1–13 in alternative order. The task is scored in terms of time to complete the items. Finally, on the “Information Processing” subtests participants are required to work through a list of items. For Part A each item comprises an array of five two digit numbers and the participants’ task is to cancel out the second highest number. On Part B each item contains a four digit array, a hyphen, and then a five digit array. The participants’ task is to cross out the number in the five digit array not contained in the four digit array. Participants are given 4 min for each task. The task is scored in terms of number of items crossed out within the time limit. A test of simple motor speed, where the participant simply has to cross out the digit ‘1’ from a page of ‘1s’ is also administered.

## 3 Results

### 3.1 Group difference analysis

Normality and heterogeneity were checked in the ASD and control groups using the Shapiro–Wilks test and Levene's test respectively. Depending on the results, group analysis was then conducted with either independent *t*-tests or Mann–Whitney tests, as appropriate. Group analysis was conducted with 77 measures derived from 17 distinct neuropsychological tests/test batteries. Where a test yields more than one sub-measure (e.g. Six Element Test yields three measures: number of tasks attempted, longest time spent on any one sub-task, and number of rule-breaks) we considered all of these sub-measures (using raw rather than scaled scores). We also considered measurements that were derived mathematically from others (e.g. Trail Making Test Part B time taken minus Part A time taken). However we did not include any measures that were sums or de facto averages of combinations of the sub-measures already considered (e.g. WAIS-III VIQ). As can be seen from Table 2 group analysis revealed limited impairment in the ASD group across the full range of tests, with only one test measure showing significant group differences at the .01 level and a further five showing significant difference at the .05 level. Observed deficits were not related to order of test administration, with the Doors and People administered at the beginning of the assessment session, the AMIPB and the Trail Making Tests administered in the middle and the Hayling towards the end of the assessment session. The remaining battery of tests revealed no significant differences between the ASD and control groups. Importantly, group analysis revealed no significant difference on the lower level tests of ability such as language comprehension, perception and short-term attention.

It is important to note that none of the significant group findings discussed above survived a Bonferroni correction at an alpha level of .05. This is not surprising in a population with marked heterogeneity of ability, consisting of both impairment and supra-normal performance. A group-level analysis would be less likely to reveal patterns of performance at the individual level that might be instructive, especially with a large battery of tests.

### 3.2 Single-case study analysis

For the multiple case series analysis, not only was each individual's performance compared with a normative sample, but also the relative levels of performance across tasks within the individual were considered. In order to conduct this analysis test scores for the 77 measures discussed above were converted to *z*-scores, based on the performance of the matched control group. The maximum range of performance on each of the 77 measures in the control group was 4.8. In the ASD group 24/77 measures (31%) showed a range exceeding this criterion set by the maximum range of the control group. As this figure is unchanged when the 6 participants who did not meet ADOS criteria are removed from the analysis we have included these participants in the remainder of the analysis in order to improve statistical power. These data quite clearly show that a defining feature of the ASD group examined here was considerable variation in performance. This variability was noted across all neuropsychological measures in our battery, including the individual WAIS-III IQ sub-measures. When summed together to produce VIQ, PIQ and FSIQ less variability was observed, although due to the underlying sub-measure variability caution needs to be applied when interpreting these summed IQ index scores. Of course, increased variance will usually be expected in a pathological group (relative to controls who are not expected to show the impairment), since an increased number will likely perform very poorly, but some will not show the characteristic under study. However the variance reported here appeared to go beyond this pattern. Some people within the ASD group were supra-normal at some tests, whilst others in the group were impaired. In other words, the increased variance for a number of measures did not derive from just impaired performance. Table 3 details those tests that showed the largest variation, and where individuals within the ASD group performed at both an impaired (<2SDs poorer than controls) and a “supra-normal” level (>2SDs above control group mean).

Nor was it especially the case that there were some people in the ASD group who were highly gifted across all tasks, and some individuals who were weak at all tasks. Many individuals showed evidence of both supra-normal performance on some tests, and impaired performance on others. There were no measures where only superior performance (>2SDs of controls) was detected in the ASD group. However there were some measures where only impaired performance was detected. For instance, six of the ASD group performed below the 1st percentile of the controls on the AMIPB Figure Recall test (immediate and delayed recall components), compared with only one control. This difference is significant at *p* = 0.046 (Fisher's Exact Test, two-tailed). None of the other measures, when submitted to such tabular analysis, reached significance individually. There are however several measures where the proportion of ASD participants who produced a noteworthy performance was greater than twice that of the control group. Thus the appropriate statistic should take the totality of this evidence into account.

To do so fully would be beyond our current knowledge of the inter-relationships and inter-dependencies between neuropsychological sub-measures. However given the size of the effects, a simplistic approach will probably suffice. Accordingly, we tallied, for each participant, the number of tasks where their performance was at least two standard deviations above or below the control mean (see Table 4). Comparison of the number of sub-measures that each individual performed at or below two standard deviations below the control mean showed a significant group difference, with the median of the ASD group (5.0) more than twice that of the controls (2.0; Mann–Whitney *U* = 134.0, *p* = 0.018). Furthermore, if one defines any performance either above or below two standard deviations of the control mean as unusual for each measure, the difference between the number of “unusual observations” for the ASD group and controls was highly significant (median ASD = 8.0; median controls = 3.0; *U* = 83.5, *p* = 0.0004). When the above analysis was replicated using the ASD group's performance as the normative standard no significant group differences were detected. This indicates that the present results cannot simply be a statistical artifact of comparing the performance of two groups with norms defined by the performance of just one of the groups.

Interestingly, this tendency towards atypicality of performance appeared to be a characteristic independent from one the most often-quoted neuropsychological features of an ASD group: motor speed. The biserial correlations between group membership (i.e. ASD or control) and performance on the neuropsychological measures showed that the strongest single predictor of group membership was the motor speed sub-measure of section B of the AMIPB Information Processing test (*r* = −.43, *p* = 0.004). However, regression analysis predicting group membership showed that motor speed accounted for a separate proportion of variance in group membership from that of the total number of atypical (or unusual) performance but that total unusual performances accounted for a larger amount of variance when these variables were considered alongside each other (Motor Speed B, *t* = −2.86, *p* = .007; total unusual performance *t* = −3.55, *p* < .001). Both variables together accounted for a remarkable 34.6% of the variance (adjusted) in group membership (*F* = 12.12, *p* < .000). Thus the prevalence of unusual or atypical performance in the ASD group cannot be attributed to some general effect of motor speed differences across the neuropsychological tests.

### 3.3 Relationship between cognition, ADOS and AQ

As can be seen from Table 1, six participants did not meet ADOS criteria. We therefore removed them from the sample and re-analysed these data. There was a limited relationship between performance on the cognitive variables and the signs and symptoms of autistic spectrum disorders. Those who showed more features of the communication problems symptomatic of ASD as measured by the ADOS tended to perform more poorly on the Doors sub-measure of the Doors and People memory test (−.552, *p* = .009). But performance on the BORB Shape Triplets subtest actually appeared to be better in those who showed these communication signs (0.567, *p* = .009). Similarly, those who showed problems on the imagination sub-scale of the ADOS performed poorly on the Proverbs test (Proverbs free inquiry *r* = −.552, *p* = 0.018; multiple choice −.50, *p* = 0.033), and on one sub-measure of the Zoo Map test (Sequencing Two *r* = −.525, *p* = .025). However they performed somewhat better on the AMIPB Information Processing test (A adjusted: *r* = 0.510, *p* = .031). Interestingly, overall individual variability in test scores across the full battery was also strongly predictive of symptoms as measured by the ADOS. Those who showed more problems on the communication sub-scale tended to show greater variability in test scores (*r* = .679, *p* < 0.001), and similarly those showing greater problems on the social sub-scale also tended to show greater variability in test scores (*r* = .549, *p* = 0.010).

There was only one neuropsychological test that showed a relationship with AQ score. This was the AMIPB Story Free Recall (Immediate Recall *r* = −.593, delayed −.588, both *p* = .005). Variability in test scores was also not related to performance on the AQ.

## 4 Discussion

We compared participants with ASD to age and IQ matched controls on a large battery of neuropsychological tests, using both group and single-case level analysis. The group-level analysis revealed a limited set of deficits on measures of processing/motor speed, a measure of executive function tapping response initiation, inhibition and set shifting and visual memory. These deficits are consistent with findings from other recent studies with adults (Ambery, Russell, Perry, Morris, & Murphy, 2006; Blair, Frith, Smith, Abell, & Cipolotti, 2002; Hill & Bird, 2006; Lopez, Lincoln, Ozonoff, & Lai, 2005; Minshew et al., 1992; Ozonoff et al., 2004; Williams, Goldstein, & Minshew, 2005). For example, Hill and Bird (2006) similarly reported deficits on the same measure of executive function tapping response initiation, inhibition and set shifting and on a measure of processing speed in their group of 22 high-functioning autism participants. However, unlike others (Hill and Bird, 2006; Lopez et al., 2005; Ozonoff et al., 2004) we did not find deficits on other measures of executive function which tap elements of planning. Our finding of impaired processing/motor speed is also consistent with their finding of generally slowed psychomotor speed in this group of participants. Similarly, our finding of impairment on tests of visual memory, but not on verbal memory, is consistent with a recent finding by Ambery et al. (2006).

No impairments were detected on the lower level tests of abilities such as short-term attention, language comprehension and perception. This supports the first hypothesis, discussed in Section 1, in other words, that impairments in higher level abilities are not based on more primary difficulties.

In support of our second hypothesis, that patterns of deficit would vary from individual to individual within the ASD group, the analysis revealed evidence of considerable variation in abilities both between ASD participants, and also within individual participants. Both sub-normal and supra-normal performance was observed within the pattern of performance for our ASD participants. In fact, the most defining feature of the ASD group was this variability, rather than sub-normal or supra-normal performance on any one particular variable or group of variables. This pattern of marked variability was not apparent from the group-level analysis, in support of our third hypothesis. Regression analysis confirmed that this atypical performance in the ASD group could not be attributed to some general effect, such as motor speed differences across the neuropsychological tests. Finally, in keeping with our fourth hypothesis, the single-case study approach also revealed evidence of individual supra-normal performance, or strengths, that again were not apparent when the data was only analysed at the group level.

### 4.1 Relationships between neuropsychological tests and diagnostic symptoms

It is also worth commenting on the findings related to autism diagnosis. The ADOS and AQ measures were included to help categorise the Autism population and not as planned independent variables. As such no specific hypothesis were formulated with regard to expected findings. However, it is interesting to note that very little relationship was observed between the ADOS, the AQ and performance on the separate neuropsychological tests. Whilst failure to find significant results may in part be due to the high variability within the data, this finding does support existing studies which have failed to find a link between Autism symptoms and neuropsychological task performance (e.g. Ozonoff et al., 2004; Pellicano, Maybery, Durkin, & Maley, 2006). We did however find a significant relationship between the amount of individual variability on tests, and the Social and Communication sub-scales from the ADOS. This raises the possibility, which would need to be confirmed with larger group numbers, that this neuropsychological variability in itself is in part characteristic of ASD and perhaps could in future serve as an important endophenotype.

### 4.2 Heterogeneity as a defining feature of high-functioning ASD, and its explanations

The observation revealed by the multiple case series analysis, showed a markedly heterogeneous pattern of performance across participants, and with differing strengths and weaknesses within individuals, poses problems for single core cognitive deficit accounts of ASD. A number of other authors (Happe et al., 2006; Willcutt et al., 2008) have similarly recently made the call for a shift away from single core deficit and simple linear models of pathways from underlying cause to symptom presentation. But what are the alternatives?

A possible alternative has previously been put forward by Minshew et al. (1997). Based on their analysis of a group of 33 ASD participants Minshew et al. (1997) hypothesized that a disorder of complex information processing leads to the multiple primary deficit syndrome that is seen in ASD. Whilst Minshew et al. do report superior performance in the results of their ASD participants, the model described in the paper is still limited by the fact that it is a deficit model, albeit a multiple primary deficit model. As such, the model of Minshew et al. does not fit well with the evidence of supra-normal performance from our study. In addition, our evidence of strong performance in some ASD participants on tasks of complex information processing is also contrary to the hypothesis that a disorder of complex information processing underlies ASD.

An alternative explanation for the findings in our study is the intra-individual variability hypothesis recently explored by Geurts et al. (2008) in their study of reaction time in ADHD, ASD and Tourette's syndrome. Geurts et al. (2008) proposed that the pattern of variability observed across and within participants may be explained by frequent lapses of attention or arousal. It is possible that these lapses could also result in a pattern of variable performance across tasks in a full cognitive battery, although such an explanation seems better suited to explaining patterns of sub-normal performance than to explaining patterns of supra-normal performance or combinations of sub-normal and supra-normal performance, as observed in our study. Further, an explanation based on variability of arousal and/or attention would predict that in addition to seeing fluctuation within and across tasks in a single testing session that we should also see fluctuations across time and testing sessions. Perhaps it could be speculated that deficits may appear when arousal levels are sub-optimal and strengths when arousal levels are optimal. But evidence of good test neurocognitive stability across time in Asperger's syndrome appears to rule out such an explanation (Nyden, Billstedt, Hjelmquist, & Gillberg, 2001), although the evidence in this respect is rather limited.

How else might simultaneous supra-performance in some domains and impairments in others be explained? One way might be if at critical developmental stages there is an alteration of the usual functional specialization of certain brain regions. Direct evidence for this hypothesis is now emerging. For example, at the physiological level, researchers have suggested that reduced synaptic pruning early in the life of an individual with ASD (Frith, 2003a) may in turn lead to an overabundance of neural pathways and encourage the formation of relatively separate populations specialized for different tasks. For example Pierce et al. (2001) reported that their ASD participants showed unique functional neural maps when processing faces, potentially as the result of aberrant developmental experience. They suggested that compared with control participants, ASD participants ‘see’ faces by utilising neural systems that are unique for each individual.

In addition, two fMRI studies by Gilbert et al. (Gilbert, Bird, Brindley, Frith, & Burgess, 2008; Gilbert et al., 2009) demonstrated unusual functional specialization within medial prefrontal cortex in ASD. Gilbert et al. (2008) demonstrated a difference in the average peak co-ordinate of activation between ASD and control groups. Moreover, Gilbert et al. (2009) showed that evidence for abnormal functional organisation within medial prefrontal cortex is more readily apparent when results are analysed on a participant-by-participant basis, rather than at the group level. In their study, the authors demonstrated that fine-grained specialization within medial prefrontal cortex, at the level of individual voxels, differed between participants with ASD and an age- and IQ-matched control group. In order to obtain this type of evidence, a single-case approach was necessary. They suggested that an abnormal process by which distinct brain regions become specialized for particular functions may lead to idiosyncratic changes in the abilities of different individuals. The present study provides behavioural data directly in line with this account. Future studies would benefit from combining the case study methodology with neuroimaging data, to provide further evidence on the relationship between changes in functional brain specialization and observations of neuropsychological heterogeneity. Approaches that seek to understand this heterogeneity offer great potential to improve our understanding of the complex pattern of brain re-organisation in ASD, which may in turn improve our understanding of the underlying causes of symptom presentation (Gilbert et al., 2009).

### 4.3 Explanations for the inconsistency of results across different studies: insights from the multiple case study approach

The limited sample sizes in this study do not allow a definitive characterization of the neuropsychological deficits in high-functioning ASD, nor even that seen within this particular set of tests. This was not the purpose of the study. But the results from the analysis at the single-case level do allow for some consideration of the plausibility of the three explanations for the inconsistency of the neuropsychological findings across different studies in the field of ASD research that were outlined in Section 1.

The first of these possibilities was that the ASD diagnosis might encompass individuals who are highly variable in terms of their abilities and disabilities, or composed of multiple distinct subgroups, which could lead to significant population sampling differences across studies. The data here is consistent with the former proposition; confirmation of the latter would require a far larger sample. But the danger of an “averaging artifact” (Shallice and Evans, 1978), where the pattern detected at a group level does not describe well any single member of the group, was highlighted well by the contrast between the striking findings at the single-case level and those much weaker ones at the group level.

The second potential explanation for the inconsistency between the findings of different studies was that the ASD diagnosis might define a group of individuals with a distinct pattern of strengths and deficits, but the deficit is such that it can cause different results according to prima facie relatively minor changes in administration or task formats. This possibility is lent support, prima facie, by the variability of performance at the single-case level. However it is challenged by the totality of the evidence: unusual performances (i.e. either supra- or sub-normal) were only seen on a limited range of the tests administered here. These ones were overwhelmingly those that had a large executive control component (including memory and perception tests with this characteristic). This seems a promising avenue for further enquiry.

The third possible explanation for inconsistency across studies is that ASD causes impairments in sensory and other processes which may then cause unpredictable impairments in tests aimed at measuring higher level tasks (e.g. problem-solving), depending on the low-level features of specific tasks (e.g. stimulus materials). This explanation was not supported at all by the present data: the ASD group were just as capable as the control group on tests of routine cognitive skills (e.g. reading, comprehension, etc.), and were matched for IQ.

### 4.4 Advantages of the multiple case study design for theoretical development

As Willcutt et al. (2008) note, in addition to explaining the heterogeneity across the spectrum of disorders such as ASD, it is also important that current models account for the significant neuropsychological heterogeneity at the level of the individual. The evidence from the present study challenges an explanation in terms of a single deficit or change in processing “style” underlying ASD. However the finding that the perturbations in cognition at the single-case level were almost confined to those tasks with a large executive component suggest a limit to the possibilities that might first be investigated. Further, they suggest that, at least in high-functioning ASD, one might first seek an explanation in terms of variable and abnormal development of a brain *system* (e.g. a multicomponential executive system), rather than a single process, or a disparate and random range of them. An investigation of that theoretical possibility is likely to require a more sophisticated approach than only reporting mean differences between groups, and it is difficult to see how this conclusion could have been reached from these data had we only done so.

## Figures and Tables

**Table 1:** Baseline characteristics of the study participants.

	ASD participants (*n* = 21)	Control participants (*n* = 22)
Age (years:months)		
Mean	31.76	30.64
Range	19–47	20–43
SD	7.73	6.31

Gender		
Males:females	17:4	18:4

IQ		
VIQ^a^		
Mean	116	117
Range	95–40	90–142
SD	12.49	12.28

PIQ^b^		
Mean	112	115
Range	86–150	95–142
SD	14.40	13.14

FSIQ^c^		
Mean	116	118
Range	95–149	91–137
SD	13.84	12.51

ADOS^d^ category		
None	6	NA
Autism spectrum	6	NA
Autism	9	NA

AQ^e^		
Mean	35.86	17.33
Range	17–48	2–42
SD	8.23	8.79

a Verbal Intelligence Quotient.

b Performance Intelligence Quotient.

c Full Scale Intelligence Quotient.

d Autism Diagnostic Observation Scale (Lord et al., 2000).

e Autism Quotient (Baron-Cohen et al., 2001).

**Table 2:** Mean and SD for ASD and control participants on those measures that showed a decrement according to a group-level analysis.

Test	ASD participants (*n* = 21)	Control participants (*n* = 22)	Sig.
Trail Making Test			
A Time	30.23 (10.29)	24.06 (7.33)	.039

AMIPB info processing^a^			
Info A motor speed	50.33 (8.22)	56.36 (8.37)	.022
Info B motor speed	51.86 (7.58)	59.68 (9.30)	.004

Hayling			
Raw 1 Time	13.10 (7.95)	7.91 (8.04)	.040

Doors and People			
Doors	19.24 (3.71)	21.23 (2.54)	.030
Visual	20.62 (4.38)	23.05 (3.47)	.039

a Adult Memory and Information Processing Battery (Coughlan et al., 1985).

**Table 3:** Neuropsychological test measures revealing variable performance in the ASD group^a^.

Measure	Range (*z*-scores)	% >2SDs^b^	% <2SDs
AMIPB Information Processing^c^			
A adjusted for speed	12.4	28.6	9.5
A total	11.3	28.6	9.5
B adjusted for speed	9.6	14.3	14.3
B total	8.7	14.3	14.3

Trail Making Test			
B time	9.8	19.0	28.6
B–A time	8.9	19.0	28.6

BORB^d^			
Drawings/drawings overlap ratio	7.0	5.0	10.0

WAIS-III^e^			
Arithmetic	6.7	28.6	19.0
Picture arrangement	6.2	9.5	14.3

a Variable performance is defined as range exceeded six standard deviations of the control performance, and there were examples of ASD performance at both <2SDs and >2SDs from the control mean.

b Percentage of the ASD group who performed at this level.

c Adult Memory and Information Processing Battery (Coughlan and Hollows, 1985).

d Birmingham Object Recognition Battery (Humphreys and Riddoch, 1993).

e Wechsler Adult Intelligence Scale—third edition (Wechsler, 1997).

**Table 4:** Evidence of extreme range of performance in the ASD participants revealed by single-case analysis^a^.

	*N*	Median	Mean	SD	Range	*p*^b^
Controls: measures supra-normal	22	0.5	0.8	1.0	0–3	.134
ASD: measures supra-normal	21	1.0	2.2	3.0	0–10	
Controls: measures impaired	22	2.0	3.2	2.8	0–9	.018
ASD: measures impaired	21	5.0	6.8	6.1	0–24	
Controls: total measures impaired	22	3.0	4.0	3.2	0–11	.0004
ASD: total measures impaired	21	8.0	9.0	5.6	2–25	

a Shown is the number of measures from the neuropsychological battery (out of a total of 77 measures) where performance were either 2SDs below the mean of the controls (“impaired”); more than 2SDs above it (“supra-normal”); or both values summed (“unusual”), with the appropriate group comparison.

b Mann–Whitney, adjusted for ties.
